# The role of pneumonia and secondary bacterial infection in fatal and serious outcomes of pandemic influenza a(H1N1)pdm09

**DOI:** 10.1186/s12879-018-3548-0

**Published:** 2018-12-07

**Authors:** Chandini Raina MacIntyre, Abrar Ahmad Chughtai, Michelle Barnes, Iman Ridda, Holly Seale, Renin Toms, Anita Heywood

**Affiliations:** 1 Biosecurity Program, The Kirby Institute, UNSW Medicine, University of New South Wales, Sydney, NSW 2052 Australia; 2 School of Public Health and Community Medicine, Faculty of Medicine, UNSW Medicine, the University of New South Wales, Samuels Building, Room 209, Sydney, NSW 2052 Australia

**Keywords:** Influenza A(H1N1)pdm09, Bacterial infection, Pneumonia, Respiratory infections hospitalization

## Abstract

**Background:**

The aim of this study was to estimate the prevalence of pneumonia and secondary bacterial infections during the pandemic of influenza A(H1N1)pdm09.

**Methods:**

A systematic review was conducted to identify relevant literature in which clinical outcomes of pandemic influenza A(H1N1)pdm09 infection were described. Published studies (between 01/01/2009 and 05/07/2012) describing cases of fatal or hospitalised A(H1N1)pdm09 and including data on bacterial testing or co-infection.

**Results:**

Seventy five studies met the inclusion criteria. Fatal cases with autopsy specimen testing were reported in 11 studies, in which any co-infection was identified in 23% of cases (*Streptococcus pneumoniae 29%)*. Eleven studies reported bacterial co-infection among hospitalised cases of A(H1N1)2009pdm with confirmed pneumonia, with a mean of 19% positive for bacteria (*Streptococcus pneumoniae 54%)*. Of 16 studies of intensive care unit (ICU) patients, bacterial co-infection identified in a mean of 19% of cases (*Streptococcus pneumoniae 26%)*. The mean prevalence of bacterial co-infection was 12% in studies of hospitalised patients not requiring ICU (*Streptococcus pneumoniae 33%)* and 16% in studies of paediatric patients hospitalised in general or pediatric intensive care unit (PICU) wards (*Streptococcus pneumoniae 16%)*.

**Conclusion:**

We found that few studies of the 2009 influenza pandemic reported on bacterial complications and testing. Of studies which did report on this, secondary bacterial infection was identified in almost one in four patients, with *Streptococcus pneumoniae* the most common bacteria identified. Bacterial complications were associated with serious outcomes such as death and admission to intensive care. Prevention and treatment of bacterial secondary infection should be an integral part of pandemic planning, and improved uptake of routine pneumococcal vaccination in adults with an indication may reduce the impact of a pandemic.

## Background

Influenza pandemics cause morbidity and mortality from both direct viral effects, which tend to present early (within the first few days), and secondary bacterial complications, which tend to present later (after the first week). Evidence of influenza predisposing to bacterial co-infection is seen in seasonal influenza epidemics, past pandemics, pathology studies and animal models [1–8]. Infection with influenza disrupts the respiratory tract by direct pathogenic effects, which then predisposes to bacterial secondary infection. Conversely, bacterial pathogens in the respiratory tract may also predispose to influenza and other viral infection [9]. During the 1918 pandemic, bacterial pneumonia was a major cause of morbidity and mortality, as shown by studies at the time as well as retrospective study of pathology specimens [10, 11]. At that time, antibiotics were not widely available as they are now, and it is thought that the high observed mortality rate was partially due to the inability to treat secondary bacterial sepsis. The most important bacterial co-infections during an influenza pandemic *S. pneumoniae*, *H. influenzae, S. aureus,* and group A *Streptococcus* (1, 4). However, two early reviews of severe cases of 2009 pandemic influenza A (H1N1) showed no evidence of bacterial pneumonia among 30 hospitalized patients with laboratory-confirmed cases in California (5) and 10 intensive-care patients in Michigan (6). These reports might have led to a perception that bacterial co-infections played a limited role or no role in pandemic influenza deaths in 2009.

The aim of this study was to estimate the prevalence of pneumonia and secondary bacterial infections during the 2009 pandemic of influenza A(H1N1)pdm09.

## Methods

### Search strategy

A systematic review was conducted according to the Preferred Reporting Items for Systematic Reviews (PRISMA) [12].We sought primary studies that presented quantitative data of invasive bacterial co-infection in influenza A(H1N1)pdm09 patients, defined as isolation of a bacterial pathogen from a sterile site. Databases searched included Medline, Pre-Medline, EmBASE and LILACS. The search strategy included a combination of Medical Subject Headings (MeSH) and text words to improve the identification of relevant publications in which bacterial co-infection was not necessarily the primary outcome of interest. The World Health Organisation (WHO) advised on the use of the standardised nomenclature influenza A(H1N1)pdm09 in October 2011 [13]. Prior to this time, various names were used to describe the pandemic virus. As such, a broad search strategy was developed to identify relevant literature in which clinical outcomes of influenza A(H1N1)pdm09 infection were described.

The Medline search included a combination of two searches. The first included the MeSH term influenza A H1N1 subtype OR text words influenza or flu adjacent to H1N1/pandemic/swine AND the MeSH term bacterial infections OR text words bacteria*, streptococcus, pneumococcus or staphylococcus adjacent to pneumonia, secondary, infection or evidence. The second search strategy included the influenza search terms and a combination of severity terms including fatal, severe, death, mortality, morbidity, hospitalisation, critical and admitted. The same search terms were applied to the other databases, after ensuring the MeSH terms of the relevant search terms were consistent across databases. Searches were limited to human studies, published in the English language between 01/01/2009 and 05/07/2012 or accessible online, ahead of print within this timeframe. Hand-searching of the reference lists of included studies and relevant reviews were also undertaken to identify other relevant papers.

### Inclusion and exclusion criteria

We included all studies of influenza A(H1N1)pdm09 which report bacterial infections (any sterile site) in influenza A(H1N1)pdm09 cases. Studies including only specific at-risk populations such as transplant or oncology patients or pregnant women were excluded. We included published English language papers of observational studies reporting on ≥10 influenza A(H1N1)pdm09 patients. Case reports and small case series of < 10 patients were also excluded.

Included cases were either fatal or hospitalised cases of confirmed or probable influenza A(H1N1)pdm09 confirmed by PCR or culture. Probable cases (13/75 studies) included those with positive influenza A serology during 2009–2010, but not testing of subtype.

However, studies which included a mixed cohort of influenza A(H1N1)pdm09 and other laboratory-confirmed influenza strains or influenza negative cases were only included if clinical outcomes could be distinguished between influenza A(H1N1)pdm09 and other confirmed strains. We excluded studies which described ambulatory influenza A(H1N1)pdm09 cases, including notifications, clinic visits or Emergency Department visits with no sub-group analysis in which hospital admission or death of patients was described.

For included studies, the definition of co-infection was broad and included any study reporting either pulmonary infection or site unspecified with or without data on bacterial type tested, or specimen tested, including those reporting negative findings. We excluded studies reporting suspected bacterial pneumonia on the basis of clinical findings alone and studies which tested specimens for presence of co-infection with respiratory viruses only. Studies reporting contamination of endotracheal tubes only and those in which patients were recruited on the basis of bacterial infection were excluded if no data on other results were reported. We present the number of cases of reported pneumonia and those requiring mechanical ventilation as per the investigators definition.

### Data extraction and assessment

**Fig. 1:**
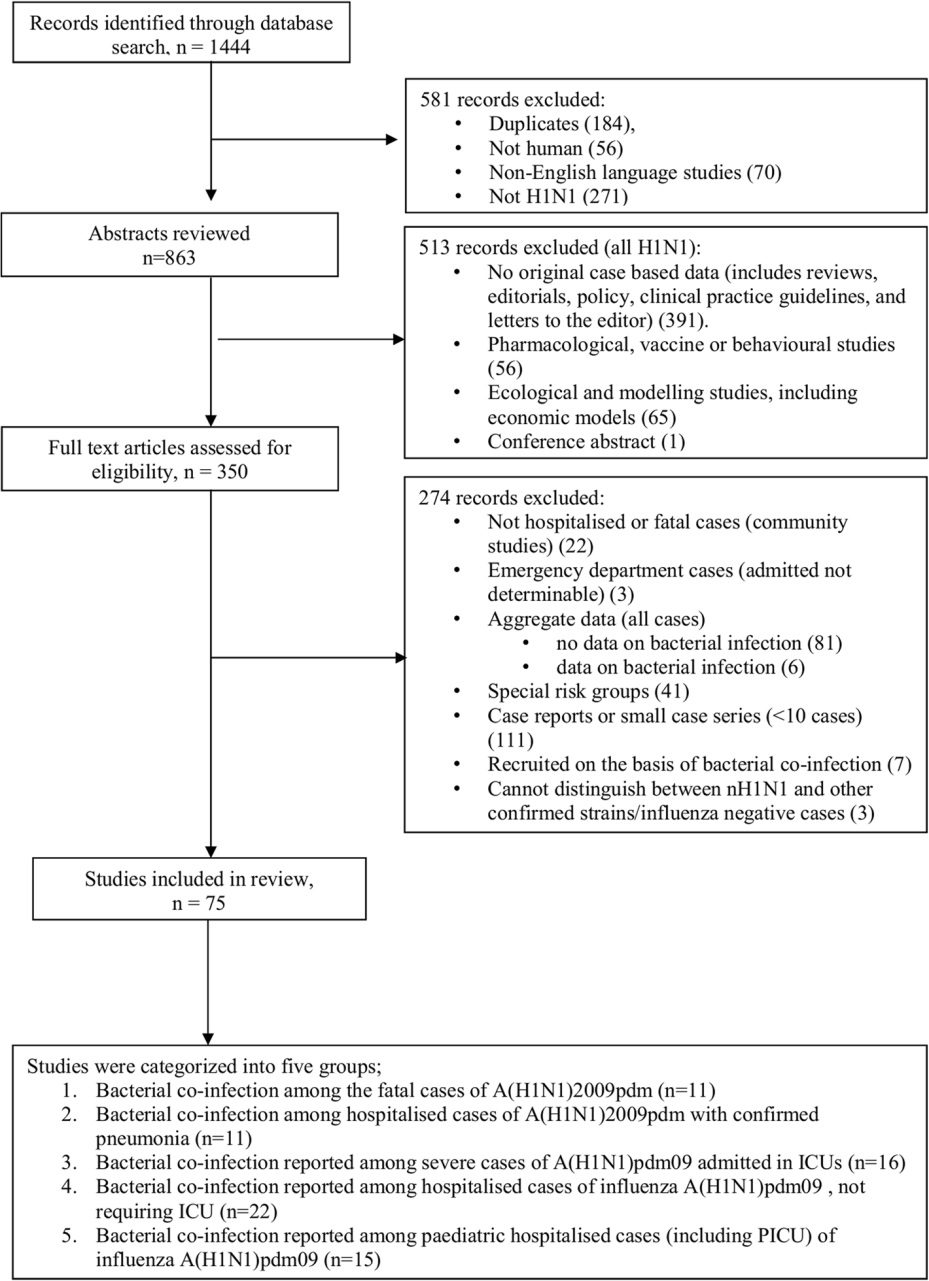
Study diagram

Five reviewers (AH, MB, RT, IR,, HS,) with experience in conducting systematic reviews independently reviewed the titles and abstracts to identify potentially relevant papers. All potentially relevant papers were read by two reviewers (AH, MB) to determine those which met the selection criteria. The results of the search strategy are shown in Fig. 1. An identical data extraction template was used by all reviewers to extract the clinical outcomes, diagnostic data and treatment. Clinical outcomes included the diagnosis of bacterial co-infection, pneumonia, and death. Treatment included mechanical ventilation and use of antibiotics. Diagnostic data included determination of pneumonia and bacterial testing. We also extracted methodological details of the relevant studies including study design, study location and methods of case ascertainment. To ensure consistency in data extraction, each study was independently data extracted by two reviewers. All findings, including discrepancies between reviewers were discussed with an independent senior reviewer (CRM).

We report bacterial findings separately from pulmonary specimens when available. When site of specimen is not specified or combined, this is reported as such. We report the percentage of tested cases positive for bacteria when available. The variability of the available data precluded the aggregation of results in a quantitative meta-analysis. Results of the studies are summarised and a critically evaluation and interpretation provided. We present results separately for fatal cases, hospitalised cases with confirmed pneumonia, cases admitted to intensive care units (ICU) and hospitalised cases admitted to general wards including criteria for admission if reported. Pneumonia, hospital admission and ICU admission were accepted according to classification in the reviewed papers.

## Results

### Summary of included studies

A total of 7845 studies were identified on the 2009 pandemic, of which 1444 articles were initially identified from our search of studies potentially about both influenza A(H1N1)pdm09 and bacterial infection. After removal of duplicates, non-human, non-English language, and non-influenza A(H1N1)pdm09 studies, 863 articles remained and abstracts were reviewed. Of those, 350 full papers were reviewed for relevance and 75 studies met the inclusion criteria. The PRISMA diagram of the study selection is shown in Fig. 1.

Reporting of patient clinical outcomes, bacterial testing and bacterial findings varied widely in the included published studies. It was not clear in many studies if pneumonia was community or hospital acquired. The studies also varied in their methodologies and proportion of patients tested, as well as reporting of bacterial testing. Samples and time of sampling were not adequately described in most of the studies.

**Fig. 2:**
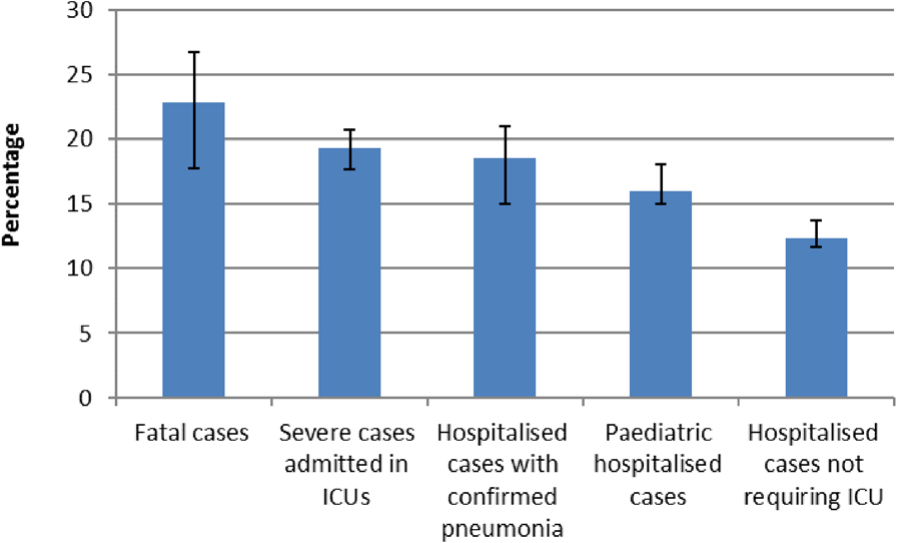
Average prevalence of bacterial infection in fatal, ICU admitted, general ward admitted and paediatric patients

Eleven studies were on fatal cases, including eight reporting autopsy results and three studies reporting bacterial findings from medical record reviews of notified deaths of confirmed influenza A(H1N1)pdm09. The remaining studies reported bacterial findings from hospitalised cases. Figure 2 shows the average prevalence of bacterial infection in fatal, ICU admitted, general ward admitted and paediatric patients.

### Bacterial co-infection among fatal cases of A(H1N1)2009pdm

Eleven studies reported evidence of bacterial co-infection of fatal confirmed cases of influenza A(H1N1)pdm09 occurring between April 2009 and May 2010 [1, 2, 14–22]. Eight studies reported autopsy results, including 8 autopsy case series [1, 2, 16–19, 21, 22] and 3 reporting bacterial findings from medical records reviews only [14, 15, 20]. Five studies were based in the USA [1, 2, 15, 16, 18] while the others were from Mexico [14], Estonia [22], Brazil [17], the United Kingdom [19], Korea [20] and Japan [21].

Influenza A(H1N1)pdm09 infection was confirmed by reverse transcriptase polymerase chain reaction (rtPCR) in either ante-mortem nasopharyngeal swab or post-mortem lung tissue specimens for all cases in all studies. Case definitions for an included fatal case reflected national surveillance reporting and/or autopsy requirements during the pandemic period and enhanced surveillance for the identification of fatal cases included the review of the death certificate registries for influenza as a cause of death.

The study details, bacterial testing and bacterial findings are summarised in Table 1. Where data were available, 44–100% of cases were hospitalised before death, including 55–100% in ICUs, with 35–100% requiring mechanical ventilation support during their hospitalisation and 25–94% of patients with clinical and/or autopsy evidence of pneumonia (viral or bacterial). From chart reviews, positive bacterial growth ranged from 2 to 38% (mean bacterial 23%) [9] of autopsied cases. Of the total coinfection cases, 29% were Streptococcus pneumoniae. The overall rate of bacterial infection was significantly higher in fatal cases compared to nonfatal cases (OR 1.71, 95% CI 1.33 to 2.20). The Korean study of standardised case reports of A(H1N1)pdm09-associated deaths identified during a period of active surveillance estimated ILI case-fatality rate to be 16 per 100,000 cases [20].

**Table 1:** Bacterial co-infection among the fatal cases of A(H1N1)2009pdm (*n* = 11)

Author (year) Study location/ period	Study type	Study population	N (%) autopsied	N (%) hospitalised prior to death	Requirement for		Antivirals^a^ n/N (%) any n/N (%) 48 h	Antibiotics^b^ n/N (%) pre n/N (%) on n/N (%) during admission	Positive bacterial growth	N (%) bacterial pneumonia	N (%) with *S.pneumoniae* Site of isolation
					ICU	Mechanical Ventilation					
Fajardo-Dolci (2009) [14] Mexico 16/3/09–16/5/09	Medical record review	*N* = 100 Consecutive notified hospitalized fatal cases	0/100 (0)	100/100 (100)	NR	84/100 (84.0)	56/100 (56) NR	94/100 (94)	2/100 (2%) (Site not mentioned)	77/82 (94.0) CXR suggestive of pneumonia.	NR
Lee (2010) [1] USA 4/09–7/09	Enhanced surveillance/confirmed cases in New York City.	*N* = 47	31/47 (66)	47/47 (100.0)	All 28 cases who died after > 24 in hospital were admitted to ICU	25/47 (80.6)	32/47 (68.0) NR	NR	NR	13/47 (27.6) By immunohistochemical analysis or PCR 21/28 with abnormal CXR and multilobar infiltrates.	8/47 (17.0) Lung/airway tissue By immunohistochemical analysis or PCR
Lucas (2010) [19] UK 4/09–1/10	Case series. 15% of reported H1N1 deaths	*N* = 68 Autopsied fatal cases	68/68 (100)	68/68 (100)	NR	NR	NR	NR	20/68 (29.4) (Culture of lung/ blood)	28/68 (41.2) - Autopsy findings - Culture and histopathology	7/68 (10.3) (6 confirmed and 1 possible through histology) isolation site Lung/ and or blood
Gill (2010) [2] USA 5/09–7/09	Case series. Autopsy request New York City (NYC) Office of chief medical examiner (*n* = 32), family requests (n = 10), deaths outside of NYC (*n* = 2)	*N* = 34 Autopsied fatal cases	34/34 (100)	21/34 (61.8)	NR	12/21 (57.1)	NR	NR	10/30 (33.3) Positive bacteria by culture, immunohistochemistry, and/or PCR	18/33 (54.4) have evidence of bacterial co-infection by tissue Gram stain.	6/30 (20) Positive for streptococcus by culture, immunohistochemistry, and/or PCR 16/33 (55) have evidence of bacterial co-infection by tissue Gram stain morphologically compatible with streptococcus.
CDC (2009) [15] USA 4/09–8/09	Multicenter case series. 100% of reported deaths	*N* = 36 Pediatric (< 18 yrs). Hospitalized fatal cases	NR	28/33 (84.8)	24/36 (66.7)	NR	19/30 (63.3) Status unknown for 6 cases 4/30 (13%)	NR	NR	10/23 (43.5) Based on culture and pathology results	3/23 (13) from multiple sites in px (BC, lung tissue, pleural fluid, CSF)
Shieh (2010) [18] USA 5/09–10/09	Notified fatal case series/US CDC	N = 100 Autopsied fatal cases	100/100 (100)	58/87 (66.7)	NR	42/57 (73.7)	44/67 (65.7) NR	NR	NR	29/100 (29) Bacterial co-infection positive through PCR and histopathology on lung tissue 38/64 (59) radiological diagnosis of pneumonia	10/100 (10) Lung tissue through PCR
CDC (2009) [16] USA 5/09–8/09	Case series (US CDC), multiple (8) states	*N* = 77 Autopsied fatal cases	77/77 (100)	8/18 (44.0)	NR	7/7 (100.0)	NR	7/9 (77.8)	NR	22/77 (28.6) Histopathology and positive PCR for bacteria	10/77 (positive through immunohistochemical assays) respiratory tissue
Mauad (2010) [17] Brazil 7/09–8/09	Case series	*N* = 21 Autopsied fatal cases	21 (100)	21 (100.0)	16/21 (76.2)	21/21 (100.0)	16/21 (76.2) NR	13/21 (61.9)	3/9 (33.3)	8/21 (38.1)	6/21 (28.6) diagnosed by culture of bronchial aspirate and/or tissue PCR
Kim (2011) [20] Korea 8/09–11/09	Active mortality inpatient surveillance	*N* = 115 Notified hospitalized fatal cases	0/115 (0)	115/115 (100%)	63/115 (54.8)	NR	100/115 (87%) 41/115 (35.6)	NR	34/115 (29.6) positive on blood or sputum culture	34/115 (29.6) positive on blood or sputum culture 97/113 (85.8) CXR suggestive of pneumonia	3/115 (2.6) bronchoalveolar lavage (BAL) Streptococcus was also isolated from blood of one case
Nakajima (2012) [21] Japan 8/09–2/10	Multicenter (15), case series, Tokyo.	*N* = 20 Autopsied fatal cases	20/20 (100)	11/20 (55.0)	NR	7/20 (35.0)	14/20 (70%) 13/20 (65%)	10/20 (50%)	4/11 (36.4%)	5/20 (25%) Based on histopathological finding (bacteria isolated in 4 of 5)	2/10 (20%) sputum, blood cultures; lung tissue
Tamme (2012) [22] Estonia 10/09–5/10	Case series	N = 21 Autopsied fatal cases	19/21 (90)	17/21 (81.0)	15/21(71.4)	NR	3/21 (14.3) 1/21 (4.8%)	16/21 (76.2)	8/21 (38.1) (Culture performed on 14 samples)	9/21 (42.8) Culture or Autopsy findings consistent with sepsis or bacterial infection	2/21 (9.5) Blood and/or lung tissue culture

Antibiotics: time started – “Pre” = started prior admission, “On” = started on admission, “During” = started during admission

*Diff* Differentiated between bacterial pneumonia, viral pneumonia and ARDS

*No diff* Did not differentiate between aetiology of abnormal chest imaging

^a^Number (percentage) of cases on antivirals; N (%) started within 48 h of symptom onset

^b^Number (percentage) of cases on antibiotics commencing pre-admission, on admission or during admission (if reported)

The lowest proportion of co-infection was reported in the first 100 confirmed deaths in Mexico [14] where 94% of patients had multiple foci of pneumonia (based on imaging) and 84% required mechanical ventilation, and only 2 cases had positive bacterial cultures (*Staphylococcus epidermidis* and *Staphylococcus hominis)*. Of the eight autopsy case series, 3 studies tested lung tissue specimens for evidence of bacterial infection in all included subjects [16–18] and identified bacterial co-infection diagnosed either before or after death in between 29 and 43% of fatal cases [16–18]. The highest proportion of culture positive bacterial co-infection from autopsy samples (38%) was reported by Tamme [22].

The reported post-mortem bacteriologic samples included culture, immunohistochemistry and PCR. No studies gave a complete picture of pulmonary bacterial co-infection during the clinical course and corresponding post-mortem findings. Bacterial co-infection identified prior to death was identified from clinically driven testing and no studies had a standardised testing protocol for all included fatal cases. On this basis, bacterial infection complicating A(H1N1)pdm09 ranged from 5 to 14% of fatal cases in the four studies [1, 17, 21, 22] reporting data from clinical testing prior to death (Table 1). Specimens obtained included sputum, bronchial aspirates and bronchoalveolar lavage, but no studies reported details of testing conducted prior to death, including the proportion tested, or the types of bacteria tested for.

### Bacterial co-infection among hospitalised cases of A(H1N1)2009pdm with confirmed pneumonia

Eleven studies reported on influenza A(H1N1)pdm09 among hospitalised cases with evidence of pneumonia and are summarised in Table 2. Pneumonia was largely defined based on radiological findings in these studies. Any positive bacterial testing was reported in 9/11 studies and positive bacterial growth was ranged from 0 to 47% (mean 19%). Streptococcus pneumoniae was the most commonly isolated pathogen (54%). In these 9 studies, *Acinobacter baumanii* was the next most commonly identified bacteria (5–21%), followed by MRSA (3–6%), *S. pneumoniae* (2–4%) and *K. Pneumonia in* (1–8%). Of the 11 studies, 2 reported no evidence of bacterial co-infection in their cohort of patients [4, 23], however, neither reported the proportion of patients tested. The study conducted in Mexico early in the pandemic [23] isolated ventilator-associated bacteria in 4 (22%) cases, with *Acinetobacter baumannii, Achromobacter xylosoxidans,* methicillin-resistant *Staphylococcus aureus,* and *Escherichia coli* identified.

**Table 2:** Bacterial co-infection among hospitalised cases of A(H1N1)2009pdm with confirmed pneumonia (*n* = 11)

Author and year	Study type	Study population	Case severity			Antivirals* n/N (%) any n/N (%) 48 h	Antibiotics† n/N (%) pre n/N (%) on n/N (%) during admission	Any positive bacterial growth	n/N (%) *S.pneumoniae* [Site of isolation]	N (%)pneumonia Method Diff/no diff
			ICU	Mechanical Ventilation	Deaths					
Perez-Padilla (2009) [23] Mexico 3/09–4/09	Single-centre case series (retrospective medical record review) of patients admitted to hospital with pneumonia and A(H1N1)pdm09 (*N* = 18)	*N* = 18	12/18 (66.7)	12/18 (66.7)	7/18 (38.9)	14/18 (77.7)	12/18 pre (66.7) 17/18 post (94.4)	0/6 (0) BC 0/2 (0) BA 0/1 (0) pleural fluid 4/18 (22.2) Ventilator Associated pneumonia	0/18 [NR] NP swab and bronchial aspirates	18 (100) CXR No diff
Chien (2010) [3] Taiwan 07/09–8/09	Nation-wide notified cases (retrospective medical record review) New pulmonary infiltrates consistent with pneumonia, compatible clinical presentations. Identification of clinicalyl significant bacteria in respiratory secretion or specimens from sterile compartments was recorded as secondary bacterial infection.	*N* = 96	35/96 (36.5)	NR	10/96 (10.4)	96/96 (100.0) - NR	NR	13/96 (13.2) pulmonary NFI (13.5)	2/99 (2) [Respiratory secretions]	13 (13.5) CXR positive
Champunot (2010) [53] Thailand 7/09–10/09	Single-centre case series (prospective); Community acquired, new pulmonary infiltrate (CXR) within 24 h of admission, clinical symptoms	*N* = 24	13/24 (54.2)	11/24 (45.8)	5/24 (8.3)	24/24 (100%) - NR	21/24 (87.5) - Pre = 6	Blood culture 0/24 (0) Sputum culture 2/24(8.3) 1/24 (4.2) [urine pneumococcal Ag TOTAL 3/24 (12.5)	1/24 (4.2) [urine pneumococcal Ag]	24 (100.0) CXR No diff
Cui (2010) [24] China 11/09–12/09	Single-centre case series (retrospective medical record review) of patients admitted to a tertiary hospital with pneumonia and H1N1(N = 68) Blood cultures (BC) - Any patient with high fever > 38.0 °C for ≥3 days or repeated fever. Sputum cultures (SC) - patients with symptoms of expectoration especially with yellowish/purulent sputum.	*N* = 68	30/68 (44)	13/68 (19.1)	10/68 (14.7)	68/68 (100.0) 50/68 (74%)	All received antibiotics 65/68 (95.6) received preadmission antibiotics	5/11 (45.5) [BC] 9/29 (31.0) [SC] Total 11/29 (37.9)	0/11 (0.0) [BC] 0/29 (0.0) [SC]	68/68 (100.0) CXR No diff
Cuquemelle [54] (2011) France 11/09–4/10	Multicenter (24) case series (retrospective)/ not having received prior antibiotics (*N* = 103) Microbiological investigations and biomarker levels were obtained as part of the routine clinical management of patients, at the discretion of the treating physician	*N* = 103	103/103 (100)	62/103 (60.2)	18/103 (17.5)	NR	0/103 (0)	48/103 (46.6) Isolation of bacteria	26/103 (25.2) [NS]	Infiltrates on all CXR
Choi [55]	Definition: the presence of an infiltrate on plain chest radiograph.	*N* = 17	17/17 (100) All in acute care unit	1/17 (5.9)	1/17 (5.9)	17/17 (100)	17/17 (100)	0/17 (0) BC 0/17 (0) SC 2/17 (11.8) urine Ag test (Legionella) 1/17 (5.9) PCR (TB)	0/17 (100) Testing for *S. pneumo*	16/17 (94.1) CXR No diff
Viasus [56]	Pneumonia was defined as the presence of a new infiltrate on a chest radiograph plus fever (temperature 38.0-C) and/or respiratory symptoms	*N* = 234 (210 tested for microbiologic studies)	53/234 (22.6)	42/234 (17.9)	12/234 (5.1)	229/234 (97.9) 50/234 (22.4)	228/234 (97.9)	36/210 (17.1) Specimens included: culture of blood, normally sterile fluids, or sputum and/or a positive urinary antigen test	26/210 (12.4)	All CXR positive
Piacentini [57]	Compares H1N1 with pneumonia in ICU and community acquired	*N* = 10	10/10 (100)	5/10 (50)	0/10 (0)	10/10 (100)	10/10 (100)	2/10 (20.0) Pre-treatment BC, SC, and urinary Ag for *S. pneumoniae* and *Legionella* sp.	2/10 (20) Specimen type NR	CXR positive (multilobar infiltrates) all except 2 (single lobe infiltrates)
Mulrennan [58]	New pulmonary infiltrates on imaging + clinical symptoms Compared with non-pneumonia H1N1	*N* = 35	11/35 (31.4)	10/35 (28.6)	2/35 (5.7)	35/35 (100)	NR	5/35 (14.3) NP, lower resp. tarct	NR	35/35 (100) CXR no diff
Ugarte (2010) [25] Chile 5/09–9/09 Adults	Multicenter (11) case series (retrospective) / adult ICU admissions Definition: positive culture from a sterile site (e.g. blood) and/or lower respiratory tract specimens, or seroconversion to atypical bacterial pathogens. LRT specimens included expectorated sputum, ET aspirated sputum and BAL	*N* = 75	75/75 (100.0))	56/75 (74.7) -	19/75 (25.3)	NR	NR	7/75 (9.3) Specimens NR	4/75 (5.3) on admission. Site NR 1/5 (20) empyema patients BC	74 (98.7) CXR No diff
Busi [4]		*N* = 40	NR	NR	1/40 (2.5)	NR	NR	0/40 (0) Specimen NR		40/40 (100) 40 had findings consistent with pneumonia. These 28/40 (70 bilaterial) Non-Diff

Antibiotics: time started – “Pre” = started prior admission, “On” = started on admission, “During” = started during admission

*Diff* Differentiated between bacterial pneumonia, viral pneumonia and ARDS;

*No diff* Did not differentiate between aetiology of abnormal chest imaging, *NR* not reported

Six studies reported use of antibiotics prior to specimen collection in subjects with bacterial co-infection in 21–22% of those tested [23, 24]. One study reported factors associated with acute respiratory distress syndrome (ARDS) or death, with the ARDS-death group more likely to have bacterial co-infections than patients who survived without ARDS or had mild disease [25]. Specimens included sputum, bronchial aspirate, pleural fluid, urine and blood with testing mainly being bacterial culture, but also multiplex PCR assay for respiratory bacterial panels (for detection of *Legionella pneumophila, Chlamydophila pneumoniae,* and *Mycoplasma pneumoniae)* and Binax NOW, an in vitro immunochromatographic assay for *Streptococcus pneumonia.* However, the mPCR assay did not test for *S. pneumoniae* in one study and the authors could not report the presence of this organism [23].

### Bacterial co-infection reported among severe cases of A(H1N1)pdm09 admitted to ICUs

Sixteen studies reported on influenza A(H1N1)pdm09 cases admitted to ICU wards and are summarised in Table 3. Criteria for admission to ICU varied in the included studies, including acute respiratory distress (ARD) [26, 27], acute respiratory failure [28, 29], required mechanical ventilation (MV) [5, 30, 31] or MV or low O2/IV vasoconstrictive drugs [32, 33], MV or ECMO [34] or admitted with no criteria provided [35–41]. Eleven studies included only PCR confirmed A(H1N1)pdm09 cases, while three included probable cases [31, 39, 40] and another three included both probable and suspected cases [5, 33, 34]. Any positive bacterial testing was reported in 12 studies and bacterial co-infection was identified in 1–43% of cases (mean bacterial 19%, Streptococcus pneumoniae 26%). One study assessed differences in mortality outcomes based on secondary bacterial pneumonia. In a large study involving admissions to 35 ICUs for ILI and ARF requiring mechanical ventilation in Argentina (*n* = 337), 24% of included patients had bacterial pneumonia on admission, 8% with *S. pneumoniae* [5]. *S.pneumoniae* co-infection was associated with higher mortality (OR 2.72 95% CI 1.05–7.06), despite concurrent antibiotic treatment on admission [5]. A Canadian study (*N* = 168) attributed secondary bacterial infection as a leading cause of death in the 29 (17.3%) fatalities that occurred in this cohort [33].

**Table 3:** Bacterial co-infection reported among severe cases of A(H1N1)pdm09 admitted in ICUs (*n* = 16)

Author and year	Study type	Study population	Case severity			Antivirals^a^ n/N (%) any n/N (%) 48 h	Antibiotics† n/N (%) pre n/N (%) on n/N (%) during admission	Any positive bacterial growth	Number (%) patients with *S.pneumoniae* and site of isolation	Number (%) with bacterial pneumonia - Method - Diff/no diff
			ICU - ECMO	Mechanical Ventilation	Deaths					
Miller (2010) [36] Utah, USA 5/09–6/09 Adults(16+)	Multicentre (4) case series (+ comparison with local resident population) / Adult (> 15 y) ICU admissions	*N* = 47	47/47 (100) - 0	13/47 (27.7) IV = 11/47 (84.6)	8/47 (14)	47/47 (100.0) - 45/47 (95.7)	44/47 (93.6) - NS	6/47 (13)	0/47 (0) BC 0/47 (0) ET aspirate 0/47 (0) SC 0/47 (0) BAL fluid	43/47(91.5) - CXR - No diff
Rello [29] (2009) Spain 6/09–7/09 Adults	Multicentre (20) case series (retrospective) / ICU adult admissions with ARF	*N* = 32	32/32 (100) - 0	24/32 (75.0) - IV = 22/32 (91.7) - NIV = 2/32 (8.3)	8/32 (25)	32/32 (100.0) -NS	32/32 (100.0)	1/32 (3.1) Secondary superinfection with Pseudomonas aeruginosa were also documented in three patients (9.3).	1/32 (3.1) aspirate 0/32 (0) BC	1/32 (3.1) respiratory culture
ANZ ECMO [34] (2009) Australia and New Zealand 6/09–8/09 All ages	Multicentre (15) cohort study (retrospective) / All ages ICU admission with ARDS treated with ECMO Includes probable cases^a^	*N* = 68	68/68 (100) - 68/68 (100)	68/68 (100)	14/68 (20.6)	64/68 (94.1) - NS	NR	19/68 (28)	10/68 (14.7) [respiratory secretion/BC]	66/68 (97.1) CXR/CT No diff
Estenssoro (2010) [5] Argentina 6/09–9/09 Adults(15+)	Multicentre (35) inception cohort study (prospective & retrospective) / adult (≥ 15 years) ICU admissions with ILI & ARF requiring MV Includes probable cases^a^	*N* = 337	337/337 (100)	337/337 (100) NIV = 64/337 (19.0)	156/337 (46.3)	328/336 (98) - NS	337/337 (100) - NS	28/337 (8.3)	28 /337 [NS] 8.3	80/337 (23.7) CXR/CT Diff
Nin [31] (2011) Chile, Uruguay 6/09–9/09	Multicenter (10) case series (> 18 yrs) (retrospective and prospective) / Respiratory failure requiring ICU mechanical ventilation ** (confirmed = 77/ 96) Includes probable cases^a^	*N* = 96	96/96 (100) 13/96 (13.5)	96/96 (100) NIV = 10/96 (10.4) IV = 86/96 (89.6) Prone ventilation = 44 /96 (45.8)HFOV = 10/96 (10.4)	48/96 (50)	84/96 (87.5) - NS	91/96 (94.8) - NS	8/96 (8)	NR	32/96 (33.3, 8 within first week of admission) - Purulent sputum, significant growth of pathogen in ET aspirate - diff
Koegelenberg (2010) [30] South Africa 8/09–9/09 Adults(18+)	Single-centre case series (prospective)/Adult (> = 18 y) ICU admissions with ARF requiring MV	*N* = 19	19/19 (100) - NR	19/19 (100) - NIV = 2/19 (10.5)	13/19 (68.4)	19/19 (100) - 14 (73.7)	NR	0/19 (19)	0/19 (0) BC 0/19 (0) ET aspirates 0/19 (0) other NS	0/19 (0) (10 cases of nosocomial infection (> = 48 h admission) - CXR
Martin-Loeches [28] (2010) Spain 1st case - 12/09 Adults (16+)	Multicentre (148) case series (prospective) /Adult (> = 15 y) ICU admissions with ARF	*N* = 645	645/645 (100)	NR - IV = 389/645 (60.3)	112/645 (17.4)	620/645 (96.1) - NS	645/645 (100) - NS	113/645 (17.5)	62 /645 (9.6) site NS Cultures routinely every day	113/645 (17.5) - CXR + pos culture - diff
Rice [39] (2012) US 4/09–4/10	Multicenter (35) case series (retrospective and prospective) / Critically ill cases (> 13 years) admitted to adult ICU’s (Confirmed = 424/683, 62%) Includes probable cases^a^	*N* = 683	683/683 (100)	231/683 (33.8) - IV = 175/683 (75.8) - NIV = 56/683 (24.2)	309/683 (45.2)	683/683 (100) -NS	NR	Total 154/683 (22.5) Sputum specimen 84/683 (12.3) Bacteraemia 50 (7.3) Both 20 (2.9)	10/683 (1.5) BC	207/683 (30.3) clinical coinfection, non-diff
CDC [27]	Patients at a tertiary care hospital in Michigan	*N* = 10	10/10 (100)	10/10 (100)	3/10 (30%)	10/10 (100)	10/10 (100)	NR	NR	NR
Kim [32]	ICU in 28 Hospitals in SK	245	245/245 (100)	162/245 (100)	99/245 (40.4)	103/245 (42)	243/245 (99.2)	91/245 (37.1)	0/245 (0)	91/245 (37.1)
Malato [35]	ICU in one hosiptal	24	24/24 (100)	6/24 (25)	4/24 (16.7)	20/20 (100)	NR	6/24 (25)	0/24 (0)	6/24 (25)
Kumar [33] (2009) Canada 4/09–8/09 All ages	Multicentre (38) cohort study (prospective & retrospective) /All age critically ill patients = ICU & requiring MV or IV medication or ≥ 60% inspired O2 fraction Includes probable cases^a^	N = 168	168/168 (100) - 7/168 (4.2)	136/168 (81.0) - IV = 128/168 (94.1) - HFOV = 20/168 (14.7)	29/168 (17.3)	NR	NR		5/168 (2.9) site NS	54/168 (32.1) possible at presentation; 41/168 (24.4) clinically dx cases following ICU admission - CXR + culture /clinical opinion
Roch [26]	ARDS cases in ICU	*N* = 18	18/18 (100)	10/18 (100)	10/10 (100)	NR	NR	0/18 (0)	0/18 (0)	0/18 (0)
Lucker [37]	One hospital ICU, medical charts reviewed	14	14/14 (100)	10/14 (71.4)	2/14 (14.3)	14/14 (100)	13/14 (92.9)	6/14 (42.8) Of ICU cases	0/14 (0)	6/14 (42.9)
Leen [38]	22 bed ICU in one hospital	*N* = 31	31/31 (100)		3/31 (10)	NR	NR	NR	NR	10/31 (32.2) Method not mentioned
Torres [40]	Hospital in Chile. Includes probable cases^a^	*N* = 11	11/11 (100)	11/11 (100)	0/11 (0)	11/11 (100)	7/11 (63.6)	1/11 (0.9) Group A Streptococcus Site NR	0/11 (0)	6/11 (54.5) Non Diff

Antibiotics: time started – “Pre” = started prior admission, “On” = started on admission, “During” = started during admission

*Diff* Differentiated between bacterial pneumonia, viral pneumonia and ARDS

*No diff* Did not differentiate between aetiology of abnormal chest imaging

^a^H1N1 testing = 53 (77.9) PCR/viral culture, 8 (11.8) serologically diagnosed but flu A not typed [34]; probable cases not defined [31]; Probable: Flu A, not otherwise subtyped [39]

### Bacterial co-infection reported among hospitalised cases of influenza A(H1N1)pdm09, not requiring ICU

Twenty two studies reported on hospitalised influenza A(H1N1)pdm09 cases (not requiring ICU) and are summarised in Table 4. Almost all studies include patients admitted to general wards, however some were transferred to ICU during the course of treatment. Most of the studies (19/22) reported bacteria testing and any positive bacterial growth was reported in 1.6–76% cases (mean bacterial 12%, Streptococcus pneumoniae 33%). The number of patients with S.pneumoniae co-infection varied from 1 to 31% depending on site of sample. Palacios et al. conducted a study in Argentina and bacteria was found in 76% of nasopharyngeal samples (152/199), of which Streptococcus pneumoniae was isolated in 31% (62/199) samples [42].

**Table 4:** Bacterial co-infection reported among hospitalised cases of influenza A(H1N1)pdm09, not requiring ICU (*n* = 22)

Author and year	Study type	Study population	Case severity n (%)			Antiviral agents - Number (%) started ≤48 h of symptoms	Antibiotics† n/N (%) pre n/N (%) on n/N (%) during admission	Any positive bacterial growth	Number (%) patients with *S.pneumoniae* and site of isolation	Number (%) with bacterial pneumonia - Method - Diff/no diff
			ICU *N*	MV - type	Deaths					
CDC (2009) [59] California, USA 4/09–5/09	State-wide passive surveillance of notified cases / Hospitalized cases for > 24 h	*N* = 30	6/30 (20)	4/30 (13.3)	0/23 (0) 7 were still hospitalised	15/30 (50) 5/30 (16.7)	0	0/100 (0)	0/100 (0)	15/25 (60) CXR Non diff
Jain [60] (2009) USA 4/09–6/09	Notified cases from 24 state health departments to CDC / Hospitalized ≥24 h	*N* = 272	67/272 (24.6)	42/272 (15.4)	19/272 (7.0)	200/268 (74.6) 78 (29.1)	206/260 (79.2) - Pre: 30/198 - on: 117/198	3/182 (1.6)	2/182 (1.1) 1 had positive lung tissue culture) 1/n urinary antigen test 1/n BAL fluid 0/n ET aspirate	- CXR 100/249 (40) (66 patients with bilateral infiltrates) 26 limited to 1 lobe, 6 limited to multiple lobes - not diff
Louie [61] (2009) California, USA 4/09–8/09	State-wide enhanced surveillance /Hospitalized or fatal all ages cases	*N* = 1088	340/1088 (31.3)	193/297 (64.9)	118/1088 (10.8)	701/884 (79.3) - 357 (40.4)	NR	46/1088 (4.2)	NR	547/833 (65.7) - CXR/CT + pos bacterial culture(s) 46 - No diff
Dhanoa [62] (2011) Malaysia 9/09–5/10	Single-centre case series (retrospective)/ Hospitalised patients all ages	*N* = 50	9/50 (18.0)	6/50 (12.0)	2/50 (4.0)	50/50 (100)	49 (98.0) - Pre = 8 - On =41	14/45 (31.1) total 45 culture samples sent	2/45 (4.4) site NR	25/50 (50) -CXR + clinical opinion - No diff
To [63] (2010) China 6/09–10/09	Single-centre case series (retrospective)/ Hospitalized adult patients	*N* = 69	28/69 (40.6)	26/69 (37.7) - NS	13/69 (18.8)	69/69 (100)	37/69 (53.6) - On = 37	0/69 (0)	0/69 (0)	25/69 (36.2) CXR Non-diff
Viasus [64] (2011) Spain 6/09–11/09	Multicentre (13) case series (prospective)/ Hospitalized ≥24 h and had a chest radiograph done	*N* = 585	71/585 (12.1)	52/585 (8.9)	13/585 (2.2)	545/585 (93.3)	416/585 (71.7)	45/585 (7.7)	28/585 (4.8)	234/585 (40) CXR non diff
Palacios (2009) [42] Argentina 6/09–7/09	Random sample specimens	*N* = 199	19/199 (9.5)	NR	20/199 (10)	96/120 (80)	14/120 (11.7)	152/199 (76.3)	62/199 (31.1)	152//199 (76) Culture pos
Chitnis [6]	Wisconsin, hospital acute care PCR + cases	*N* = 252	59/252 (23.4)	34/59 (58) Of tho9se cases admitted to ICU	11/252 (4.3)	215/250 (86)	204/249 (81.9)	19/241 (7.9)	NR	123/229 (53.7) CXR non diff
Riera [65]	13 Hospitals in Spain	*N* = 585	71/585 (12.1)	52/585 (8.9)	13/585 (2.2)	545/585 (93.1) 202/585 (34.5)	316/585 (54)	45/585 (7.7) (Sputum)	2/585 (0.3) (1 sputum and 1 antigenuria positive	CXR infil 234/585 (40) Multilobarl infilt 135/585 (23.1)
Semionov [66]	147 cases with CXR results available in Montreal	*N* = 147	8/147 (5.4)	6/147 (4.1)	4/147 (2.7)	NR	NR	21/42 (50) (of 42 radiological positive cases)	5/21 (23.8) (of 21 positive bacterial cases)	42/147 (28.6) cases positive CXR Non diff
To [67]	74 cases in Hong Kong	*N* = 74	28/74 (37.8)	26/74 (35.1)	2/74 (2.7)	69/74 (93.2)	52/74 (70.3)	9/74 (12.2) bacterial positive 8 sputum 1 blood	0/74 (0)	9/74 (12.2) bacterial positive 8 sputum 1 blood Diff
Masia [68]	Complicated hospital admitted cases in Spain > 18 years, out patients	*N* = 100	4/100 (4)	NR	0/100 (0)	NR	NR	14/100 (14)	14/100 (14) Diff 8 urinary antigen pos, 2 isolated from blood and 4 from sputum	14/100 (14) Diff
Pecavar [69]	Hospitalized cases	*N* = 66	7/66 (10.6)	NR	NR	62/64 (96.9)	35/61 (57.4)	5/63 (7.9)	2/63 (3.2)	29/57 (50.9) CXR Non diff
Liu [70]	One hospital in China	*N* = 46	NR	NR	NR	46/46 (100)	9/46 (19.6)	9/15 (60)	NR	44/46 (95.6) CXR or CT scan abnormal Non diff
Nguyen (2010) [71] UK 4/09–9/09	Multicentre (55) case series (retrospective)/ Hospitalized cases	*N* = 631	53/631 (8.4)	NR - IV = 21/631 (3.3)	29/631 (4.6)	474/631 (75.1) - NS	366/631 (58)	4/102 (3.9%) of cases with radiological pneumonia	1/102 (0.9) sputum culture 0/102 (0) BC	102/349 (29.2) - CXR - No diff
Santa-Olalla Peralta [72] (2010) Spain 4/09–12/09	National surveillance of severe cases (retrospective) / Hospitalized patients all ages (, in Spain	*N* = 3025	852/3025 (28.2)	438/3.25 (14.5)	200/3025 (6.6)	2521/2779 (90.7) - 711/2020 (35.2)	NR	292/957 (30.5, bacteria isolated not reported)	NR	NR
Venkata [73] (2010) USA 5/09–12/09	Single-centre case series (retrospective)/ Electronic medical records of hospitalized adult patients	N = 66	29/66 (43.9)	23/66 (34.8) - IV = 17 (73.9) - NIV = 6 (26.1)	5/66 (7.6) 4 more died after discharge from hospital	60/66 (90.9) - NR		14/ 29 (48.2) Bac culture pos	3/29 (10.3), site NS	14/29 (48.2) confirmed and 10/29 (34.5) probable bacterial pneumonia - NR - Diff
Jartti [74]	Cases with severe cases and CXR finding available, I hospital in Finland	*N* = 135	18/135 (13%)	18/135 (13.3)	3/135 (2.2)	NR	NR	5/135 (3.7) Site not mentioned	1 /135 (0.7) Isolated from Plural Fluid	84/135 (62.2)
Rizzo [75]	Sentinel sites, Italy	*N* = 1278	NR	NR	NR	NR	NR	33/1278 (2.6)	0/1278 (0)	271/1278 (21.2) Non-diff
Kopel [76]	Til Aviv, cases in ICU and PICU	N = 17	17/17 (100)	NR	7/17 (41.2)	NR	NR	9/17 cases (52.9) (but likely nosocomial infection, so not included)	0/17 (0)	NR
D’Ortenzio [41]	ReUnion Island, all sites, included 785 reported cases, 282 hospitalized cases included here	*N* = 282	24/282 (8.5)	15/282 (5.3)	7/282 (2.5)	92/171 (53.8) 39/163 (23.9) (within 48 h)	NR	NR	NR	24/83 (28.9) (Confirmed and suspected) Sample not reported
Dominguez-Cherit [77]	Critically ill, hospitalized in 6 hospital in Maxico	N = 58	58/58 (100)	54/58 (93.1)	24/ 58 (41.4)	57/58 (98.3)	52/58 (89.6)	4/58 (6.9)	0/58 (0)	NR

Antibiotics: time started – “Pre” = started prior admission, “On” = started on admission, “During” = started during admission

*Diff* Differentiated between bacterial pneumonia, viral pneumonia and ARDS

*No diff* Did not differentiate between aetiology of abnormal chest imaging

### Bacterial co-infection reported among paediatric hospitalised cases (including PICU) of influenza A(H1N1)pdm09

Fifteen studies reported on admitted paediatric influenza A(H1N1)pdm09 cases, including 11 to any hospital ward and 6 restricted to PICU and are summarised in Table 5. The mean prevalence of bacterial co-infection was 16% in studies of paediatric patients hospitalised in general or pediatric intensive care unit (PICU) wards. Rates of bacterial co-infections vary in these studies, ranging from 0 to 87% (mean 5%) in any hospital ward admission to 13–34% (mean 32%) in admission to PICU. The highest rate was reported by Okada [43] who conducted a study in Japan on 46 hospitalised children from July 2009 to January 2010. Bacteria were isolated from nasopharyngeal swabs of 87% admitted cases (40/46)- S. pneumoniae 37.0%; S. pneumoniae and H. influenzae 23.9%, H. influenza, 26.1% and *S. aureus* 23.9%.

**Table 5:** Bacterial co-infection reported among paediatric hospitalised cases (including PICU) of influenza A(H1N1)pdm09 (*n* = 15)

Author and year	Study type	Diagnosis of influenza/ cases	Antiviral agents - Number (%) started ≤48 h of symptoms	Number (%) patients with *S.pneumoniae* and site of isolation	Any bacteria positive	Number (%) with bacterial pneumonia - Method - Diff/no diff	Antibiotics used (Pre, on, during)	Required		
								ICU - ECMO	MV	Deaths
Hospitalised										
Louie (2010) [7] California USA 4/09–8/09	State-wide surveillance (California) / Hospitalized or died cases (< 18 years)	PCR/345	221/345 (64.1) 88/ 345 (25.5) with in 48 h	3/345 (0.9) isolation site NR	15/345 (4.3)	138/229 (60.3) (F: 4/5; H: 134/224) 163/278 (CT + CXR pos) - No diff	NR	94/345 (27.4)	35/94 (37) in text	9/345 (2.6)
Libster [78] (2010) Argentina 5/09–7/09	Multicentre (6) case series (retrospective)/ Hospitalized cases (< 18 years)	PCR/ 251	- 22/ 171(12.9) with in 48 h (4/34 PICU Px, 18/137 ward px)	2/121 (1.7) BC 1/4 (25.0) empyema	10 /121 (8) Blood culture	25/251 (10.0) bacterial confirm - Among 92 CXR, 78% diagnosis was pneumonia -NonDiff	186/251 (74.1) - On = 82	47/251 (18.7)	42/251 (16.7)	13/251 (5.2)
Okada [43] 2011 Japan 7/09–1/10	Single-centre case series (retrospective) / Pneumonia, pharyngitis or bronchitis cases (< 15 years) (n = unclear).	PCR/46	44/46 (95.6) - NR	28/46 (60.9) NP	40/46 (86.9) NP positive swab	40/46 (86.9%) NP swab Bac pneumonia 21/46 (45.6%) unilateral infiltrates - CXR - No diff	32/46 (69.6) - NS	NR	NR	0/46 (0)
Kumar [79] (2010) Wisconsin, USA 4/09–8/09	Single-centre case series (retrospective record review)/ Hospitalized (> = 24 h) cases (< 19 years)	PCR/75	74/75 (98.7) - NR	0 /75 (0)	0/75 (0)	23/75 (34.3) - CXR - Diff	60/75 (80.0) - Pre = 12 - On/during = 48	14/75 (18.7)	4/75 (5.3)	2/75 (2.7)
Miroballi [80] (2010) New York, USA 05/09–07/09	Multicentre (2) case series (retrospective) / Hospitalized cases (< 18)	PCR (54), EIA, DFA, viral culture/ 115	97/115 (84.3) - NR	2/115 (1.7) BC 1/115 (0.9) respiratory secretions	4/115 (3.5)	Total NR (11/35 in PICU) - NR - Diff NS	89/115 (77.4) - NS	35/115 (30.4)	11/115 (9.6) in PICU	1/115 (0.9)
O’Riordan [81]	Retrospective case review/hospitalised cases (< 18 yrs)	PCR/ 58	12/58 (20.7) - NR	1/58 (1.7) BC	1/58 (1.7) bacterial culture positive	17/58 (29.3) - CXR - No diff	56/58 (96.6) - NS	12/58 (20.7)	7/58 (12.1)	0/58 (0)
Bettinger [82] (2010) Canada 5/09–8/09	National active surveillance / hospitalized cases (< 17 years)	PCR/235	107/235 (45.5) - NR	3/235 (1.3) isolation site NR	8/235 (3.4) culture positive	8/235 (3.4) culture positive	203/235 (86.4) - NS	39/235 (16.6)	15/235 (6.4)	2/235 (0.9)
Lockman [83] 2010	Retrospective case review (notes)/cases admitted to ICU	PCR/ 13	11/13 (84.6) - 5 (45.5)	0/13 (0)	0/13 (0)	3/13 (23%) Nondiff CXR	3/13 (23.1) - NS	13/13 (100)	6/13 (46.2) - IV =4 (66.7) - NIV = 2 (33.3)	0/13 (0)
Stein [84]	< 18 year, ARI cases, hospitalized in 7 medical centers in Israel	PCR/ 478	413/478 (87.1)	2/478 (0.4)	4/478 (0.8)	172/478 (35.9) CXR non-diff	215/478 (45)	42/478 (8.8)	15/478 (3.1)	3/478 (0.6%)
Tamma [85]	1 hospital, lab confirm US, < 18 years, retrospective cohot in US	PCR/ 133	106/133 (79.7) 51/133 < 48 h	0/133 (0)	0/133 (0)	28/122 (22.9) CXR nondiff	61/85 (72%)	27 (20.3)	11/133 (8%)	0
Parakh [86]	1 hospital, retrospective record review, India PICU and ICU, cases with age < 18 with ILI	PCR/ 25	25/25 (100)	0/25 (0)	0/25 (0)	8/25 (32) CXR nondiff	NR	7/25 (28)	4/25 (16)	3/25 (12)
PICU										
Farias [87]	Prospective multicentred study/includes nosocomial cases in Argentina(*n* = 147)	PCR/ 147	135/147 (91.8) - 116/147 (85.9)	6/147 (4) isolation site NR	50/147 (34)	NR	143/147 (97.3)	147/147 (100)	139/147 (94.6) - IV = 117 (84.2) - NIV = 22 (15.8)	57/147 (38.8)
Randolph (2011) [88] USA 4/09–4/10	Multicentre (35) case series (retrospective & prospective). Probable 293/838 (35%) cases PICU cases (< 21 years)	Confirmed- PCR, viral culture Probable- direct fluorescent antibody, rapid influenza diagnostic test (*n* = 838)	751/838 (89.6) - NR (before ICU admission =49)	0/38 (0) BC 15/274 (5.5) respiratory secretions BC: 0	274/838 (32.7) had pneumonia/other bacterial co-infection From resp. secretions	274/838 (32.7) had pneumonia/other bacterial co-infection From resp. secretions	NR	838/838 (100) - 33 (3.9)	546/838 (67.3)	75/838 (8.9)
Shin [89]	South Korea, critically ill cases	PCR/30	29/30 (96.7)	0/30 (0)	4/30 (13.3) Blood c/s positive	22/29 (75.9) CXR non diff	NR	30/30 (0)	16/30 (53.3)	14//30 (46.7)
Jouvet [8]	Canada PICUs,	PCR or culture positive /57	44/57 (77.2)	5/27 (18.5) Lower resp. samples	12/57 (21.1) Lower resp. samples	12/57 (21.1) Lower resp. samples	54/57 (94.7)	2/57 (3.5)	39/57 (68.4)	4/57 (7)

Antibiotics: time started – “Pre” = started prior admission, “On” = started on admission, “During” = started during admission

*Diff* Differentiated between bacterial pneumonia, viral pneumonia and ARDS

*No diff* Did not differentiate between aetiology of abnormal chest imaging

## Discussion

Secondary bacterial infection was an important complication of the 2009 influenza pandemic, with almost 1 in 4 severe or fatal cases having bacterial secondary infections, albeit with varying rates. Bacterial infection appeared to be associated with morbidity and mortality, with higher rates in adults, ICU patients and those with a fatal outcome. *Streptococcus pneumoniae* was the most common bacteria identified, and in ICU patients, ventilator associated pneumonia with organisms such as *Acinetobacter baumannii, Achromobacter xylosoxidans,* methicillin-resistant *Staphylococcus aureus,* and *Escherichia coli* was common. The prevalence of bacterial co-infection was lower in studies of hospitalized patients not requiring ICU and in studies of paediatric hospitalized patients, although the latter was quite varied.

The overall morbidity and mortality of the 2009 pandemic varied by country, but was cited as being similar to a severe seasonal influenza epidemic [44]. However, two important differences in the epidemiologic pattern of the 2009 pandemic were firstly, a low average age of death in fatal cases (53 years compared to 83 years during seasonal influenza) and high intensive care unit (ICU) occupancy rates [45]. These two features hint at a severe population impact, and a UK study showed a “w” shaped morbidity curve with a peak in young adults [46].

The 1918 pandemic has served as a reference point in pandemic planning, but availability of antibiotics, critical care and extra-corporeal membrane oxygenation (ECMO) have vastly improved survival during a contemporary pandemic, so it would be unlikely that case fatality rates of 1918 would recur in the modern era [45]. The use of ECMO rose sharply in 2009 and is associated with high rates of survival [47].

Further, in understanding the morbidity and mortality impact of a modern pandemic, it is important to quantify the relative contribution of direct viral effects compared to bacterial secondary infections, as treatment and prevention options are also available for bacterial infections.

Testing for bacterial complications during an influenza pandemic is important, but was neglected in most studies which we screened for this review. For optimal response and mitigation of preventable morbidity and mortality, active surveillance during both seasonal and pandemic influenza is necessary, and systems should be in place for rapid assessment of secondary bacterial morbidity and mortality. Diagnosis and treatment of secondary bacterial infections should always be considered during a pandemic [10].

Currently there are limited data on bacterial coinfection during influenza pandemic in 1918. Morens et al. reviewed autopsy data from 58 lung tissue samples collected during the 1918 influenza pandemic and histologic evidence of severe bacterial pneumonia was found in almost all samples [10]. The authors also did a literature search around autopsy case series and examined data of 3074 subjects in 68 high quality autopsy case series. This showed that more than 92% of autopsy lung cultures were positive for at least one bacterium [10]. Another study by Chien et al. reviewed the studies that reported more than 10 sterile-site antemortem cultures from adults with pneumonia during 1918 pandemic [48]. Culture positivity rates among influenza cases without pneumonia was very low (mean < 1%), compare to those with pneumonia (mean, 16%; range, 2 to 50) [48]. Bacterial co-infection rates among hospitalised cases with confirmed pneumonia in this study was 19%, which is comparable to Chien et al.

The rate of bacterial co-infection may be underestimated as many cases are not tested for bacterial infections, and bacterial pneumonia cannot always be differentiated from viral pneumonia on the basis of clinical presentation, radiology and routine blood tests. There is also a need to develop diagnostic algorithms for early identification of bacterial infections in these cases to ensure early detection and treatment of bacterial complications.

The WHO guidelines on vaccines and antivirals for a pandemic, along with many country-specific pandemic plans, do not consider pneumococcal vaccines [49]. The CAPITA trial shows efficacy of conjugate pneumococcal vaccine against pneumonia [50], and the polysaccharide vaccine also has efficacy against invasive pneumococcal disease [51]. We have found evidence that severe and fatal cases of influenza during the 2009 pandemic did comprise secondary bacterial causes, including *streptococcus pneumoniae* as a contributing factor. Vaccination against streptococcus pneumonia is often neglected in pandemic planning [52], but could have a positive impact on morbidity and mortality. The evidence confirms that prevention of bacterial secondary infection should be an integral part of pandemic planning. Improving uptake of routine pneumococcal vaccination in adults with an indication will cover most patients at risk, and may reduce the impact of a pandemic.

To our knowledge, this is the first systematic review to estimate the prevalence of pneumonia and secondary bacterial infections during pandemic influenza A(H1N1)pdm09. We calculated bacterial co-infection rates separately for fatal cases, hospitalised cases with confirmed pneumonia, hospitalised cases admitted to ICU, hospitalised cases admitted to general wards and paediatric hospitalised cases, showing the highest risk of bacterial infection for fatal and ICU admitted cases.

## Conclusion

We found that secondary bacterial infection was an important complication of the 2009 influenza pandemic, with *Streptococcus pneumoniae* the most common bacteria identified. Bacterial infection appeared to be associated with morbidity and mortality, with higher rates in adults, ICU patients and those with a fatal outcome. Prevention and treatment of bacterial secondary infection should be an integral part of pandemic planning, and improved uptake of routine pneumococcal vaccination in adults with an indication may reduce the impact of a pandemic.

## Abbreviations

ARDS: Acute respiratory distress syndrome
ARTI: Acute respiratory tract infection
BAL: Bronchoalveolar lavage
BAL: Broncho-alveolar lavage
BC: Blood culture
CT: Computerised tomography
CXR: Chest x-ray
ET: Endotreacheal tube
ICU: Intensive care unit
ILI: Influenza like illness
IV: Invasive ventilation
MeSH: Medical Subject Headings
MV: Mechanical ventilation
NIV: Noninvasive ventilation
NR: Not reported
NS: Not specified
PICU: Pediatric intensive care unit
PRISMA: Preferred Reporting Items for Systematic Reviews
SC: Sputum culture
